# Immune Characteristics of IgA Nephropathy With Minimal Change Disease

**DOI:** 10.3389/fphar.2021.793511

**Published:** 2021-12-16

**Authors:** Huixian Li, Wanhong Lu, Haiyun Li, Xiaoling Liu, Xue Zhang, Liyi Xie, Ping Lan, Xiaoyang Yu, Yinjuan Dai, Xinfang Xie, Jicheng Lv

**Affiliations:** ^1^ Department of Nephrology, The First Affiliated Hospital of Xi’an Jiaotong University, Xi’an, China; ^2^ MOE Key Laboratory of Environment and Genes Related to Diseases, School of Basic Medical Sciences, Xi’an Jiaotong University, Xi’an, China; ^3^ MOE Key Laboratory of cell Activities and Stress Adaptations, School of Life Science, Lanzhou University, Lanzhou, China; ^4^ Renal Division, Department of Medicine, Peking University First Hospital, Beijing, China; ^5^ Institute of Nephrology, Peking University, Beijing, China

**Keywords:** MCD-IgAN, galactose deficient IgA1, anti-glycan autoantibodies, inflammation, IgA nephropathy, minimal change disease

## Abstract

**Background:** IgA nephropathy (IgAN) has a high degree of heterogeneity in clinical and pathological features. Among all subsets of IgAN, the pathogenesis of IgAN with minimal change disease (MCD-IgAN) remained controversial.

**Methods:** We analyzed the clinical and pathological characteristics of MCD-IgAN patients in a retrospective cohort. Patients diagnosed with IgAN, excluding MCD-IgAN, were randomly selected as controls. Levels of plasma galactose-deficient IgA1 (GdIgA1), IgG autoantibodies against GdIgA1, GdIgA1 deposition in the glomerulus, and inflammatory reactivity of circulating poly-IgA1 complexes to cultured mesangial cells were evaluated.

**Results:** Patients with MCD-IgAN had significantly higher levels of proteinuria and estimated glomerular filtration rate (eGFR), lower levels of albumin and urine blood cells, and milder histological lesions by a light microscope compared to IgAN patients, which bears a resemblance to MCD. Lower levels of GdIgA1 (3.41 ± 1.68 vs. 4.92 ± 2.30 μg/ml, *p* = 0.009) and IgG antiglycan autoantibodies (23.25 ± 22.59 vs. 76.58 ± 71.22 IU/ml, *p* < 0.001) were found in MCD-IgAN patients than those in IgAN controls. Meanwhile, weaker fluorescence intensities of both IgA and GdIgA1 were observed in the glomerulus of MCD-IgAN patients compared to those in IgAN patients. Furthermore, poly-IgA1 complexes from MCD-IgAN patients induced weaker inflammatory effects on cultured mesangial cells than those from IgAN patients *in vitro*.

**Conclusion:** The results demonstrated that MCD-IgAN cases represent a dual glomerulopathy, namely, mild IgAN with superimposed MCD, which furthermore provides substantial evidence for the corticosteroids therapy in MCD-IgAN patients as the guidelines recommended.

## Introduction

IgA nephropathy (IgAN) is one of the most common glomerulonephritis worldwide, especially in Asia (Wyatt and Julian, 2013). Approximately 30%–40% of patients suffered a slow but relentless clinical course that could progress to end-stage kidney disease (ESKD) for 20–30 years (Lai et al., 2016; D’Amico 2004). In clinical practice, IgAN is diagnosed as dominant IgA deposition in the mesangial area by kidney biopsy. However, the clinical and pathological manifestations of IgAN are diverse (Zhang and Barratt, 2021). The clinical course of the disease ranges from isolated hematuria, subnephrotic proteinuria, nephrotic proteinuria to rapidly progressive renal failure, and kidney biopsy findings vary from mild mesangial proliferation to diffuse crescent formation, suggesting those might not be the same disease (Lai et al., 2016). Among all subsets of IgAN, cases presenting nephrotic syndrome (NS) and mild mesangial proliferation are rare, accounting for approximately 5%–10% of all IgAN patients (Barratt and Feehally, 2006; Kim et al., 2012). Therefore, this variant form of IgAN with clinical NS presentation and electron microscope (EM) features of diffuse foot process effacement resembling minimal change disease (MCD) is defined as MCD-IgAN (Floege et al., 2019).

In general, there is a reasonable correlation between clinical and pathological findings in IgAN; mounting studies indicate that heavy proteinuria at baseline is associated with more aggressive disease accompanied by severe glomerular damage or renal insufficiency (Thompson et al., 2019). While previous studies revealed the clinical features, podocytopathic variant and the good response to glucocorticoids in these MCD-IgAN patients are more consistent with MCD patients (Westhoff et al., 2006; Kim et al., 2009; Qin et al., 2013; Wang et al., 2013; Herlitz et al., 2014; Li et al., 2016). Therefore, it remained controversial whether these patients suffered from a specific podocytopathic variant type of IgAN, or the existence of MCD in mild IgAN, or even MCD with non-pathogenic IgA deposition.

Recent studies have indicated four crucial processes contributing to IgAN development (Suzuki et al., 2011). The first two established processes are the existence of higher levels of galactose-deficient IgA1 (GdIgA1) and autoantibodies against GdIgA1 in circulation (Tomana et al., 1999; Moldoveanu et al., 2007; Suzuki et al., 2009; Zhao et al., 2012). Then, poly-IgA1 complexes composed of GdIgA1(Allen et al., 2001) and its autoantibodies (Rizk et al., 2019) accumulate in the renal mesangium. The deposits finally induce mesangial proliferation, expansion of extracellular matrix, and secretion of cytokines and chemokines, resulting in renal injury (Novak et al., 2005). Until now, as a specific subset of IgAN, whether the poly-IgA1 complexes in MCD-IgAN are coincidental or playing the same pathophysiological role as in IgAN remains unclear. In this study, we examined the immune features, including levels of circulating GdIgA1, antiglycan IgG autoantibodies, renal deposits of GdIgA1, and the inflammatory effects of poly-IgA1 complexes in IgAN-MCD to elucidate it.

## Materials and Methods

### Patients and Healthy Controls

IgAN was diagnosed by immunofluorescence showing IgA as the dominant or co-dominant immunoglobulin in the mesangial deposits and the deposition of electron-dense materials in mesangium on ultrastructural examination in the absence of secondary causes. A total of 656 patients were diagnosed as IgAN patients from January 2018 to November 2020 at the First Affiliated Hospital of Xi’an Jiaotong University, of whom 27 patients with 1) diffuse podocyte foot process effacement at electron microscopy (>50% of the capillary surface area involved); 2) mild mesangial hypercellularity; 3) without endocapillary proliferation, segmental glomerulosclerosis, interstitial fibrosis and tubular atrophy, or cellular crescents were diagnosed with MCD-IgAN (Supplementary Figure S1). Sixty-eight IgAN patients without nephrotic proteinuria during the same periods were randomly selected as controls at the ratio of 1:2 approximately. In the same period, age- and gender-matched healthy physical examinees were chosen randomly. Oxford Classification scores were performed for all biopsy specimens by an experienced renal pathologist. The extent of podocyte foot process effacement was evaluated under transmission electron microscopy (TEM). Clinical and pathological characteristics of all patients were collected at the time of renal biopsy. MCD-IgAN patients were followed until the remission of proteinuria. Complete remission (CR) was defined as 24-h urine protein <500 mg/day. This study was conducted in adherence to the Declaration of Helsinki and was approved by the medical ethics committee at the First Affiliated Hospital of Xi’an Jiaotong University. Plasma and urine samples from patients at the time of renal biopsy and from thirty-two age- and gender-matched healthy individuals were stored at −80°C before use.

### Plasma IgA1, Gd-IgA1, and IgG Autoantibodies Targeting the Gd-IgA1

The levels of plasma IgA1 were detected by enzyme-linked immunosorbent assay (ELISA) with a previously established protocol (Zhang et al., 2019). Plasma Gd-IgA1 levels were quantified by using the Gd-IgA1-specific monoclonal antibody KM55 ELISA Kit (IBL, Naka, Japan) according to the suggested procedure. See the detailed methods in supplementary materials.

Circulating autoantibodies of IgG against GdIgA1 were detected according to our previous protocol (Wang et al., 2021). Briefly, IgA1 F(ab)_2_ with hinge region of its heavy chain, namely, F(ab)_2_-HR, was used as antigen. After blocking all wells with 1% bovine serum albumin (BSA)/phosphate-buffered saline (PBS), diluted plasma (1:100) and standards were added to each well; alkaline phosphatase-conjugated goat antihuman IgG monoclonal antibody (Sigma, United States) was used for detection. Detailed methods were described in supplementary materials.

### Immunofluorescent Staining of Gd-IgA1 and IgA in Kidney

Formalin-fixed paraffin-embedded (FFPE) tissues were sectioned at 3 μm for IgA and GdIgA1 staining. Renal slides from MCD, membranous nephropathy, and healthy renal allograft were used as negative controls. Antigen retrieval was performed with 0.05% protease from *Bacillus licheniformis* (Sigma-Aldrich, St. Louis, MO, United States) at room temperature for 2 h. Then, 3% bovine serum albumin in phosphate-buffered saline was used to block the non-specific binding. Next, the slides were incubated with rat antihuman GdIgA1 antibody (Immuno-Biological Laboratories, Japan) for 1 h at 37°C followed by Alexa Fluor 555-conjugated goat antirat IgG antibody (Abcam, United States) and fluorescein isothiocyanate (FITC)-conjugated polyclonal rabbit antihuman IgA antibody (Dako, Japan) incubation at 37°C for 30 min. Immunofluorescence images were captured using a Nikon microscope 90i (Nikon Instruments Inc., Japan). Glomerular staining of IgA and Gd-IgA1 was evaluated by the Image Pro Plus analysis software version 6.0. Semiquantitative analysis of fluorescence intensity for IgA or GdIgA1 staining was described as the glomerular mean optical density (integrated option density/glomerular area). At least eight fields of glomerular vision per kidney section were randomly captured at 400× for semiquantitative assessment of immunofluorescent staining.

### Poly-IgA1 Complex Purification and Mesangial Stimulation

Ten milliliters of peripheral venous blood (EDTA anticoagulated) was collected at the renal biopsy from recruited eight patients with MCD-IgAN, seven patients with IgAN, and five patients with MCD. Poly-IgA1 complexes were separately isolated from the plasma sample of each patient by jacalin affinity chromatography and then Sephacryl S-300 gel filtration chromatography, as previously reported (Zhu et al., 2013). Primary human mesangial cells (HMCs) (ScienCellTM, Carlsbad, CA, United States) were cultured to the sixth passage with mesangial cell medium (MCM) containing mesangial cell growth supplement (MsCGS), 2.5% FBS. After overnight starvation with mesangial cell medium containing 0.05% FBS and 0% mesangial cell growth supplement, HMCs were stimulated and cultured with 100 μg/ml poly-IgA1 in MCM medium for 48 h. The supernatants were collected after being centrifuged and stored at −80°C for cytokine detection. IL-6 and MCP-1 levels in cell culture supernatants after poly-IgA1 treatment, as well as urinary MCP-1 in patients and healthy controls were detected by using ELISA kit (4A Biotech Co., Ltd., Beijing).

### Statistical Analyses

Non-normally distributed and normally distributed quantitative parameters were expressed as medians (IQRs) and means ± standard deviations, respectively. Kolmogorov–Smirnov test was performed to determine normal distributions of quantitative parameters. Categorical data were represented by frequencies or ratios. Differences in continuous variables were assessed using the non-parametric test (Mann–Whitney *U* test) and t-test when the data were not normally distributed or normally distributed, respectively. Kaplan–Meier analysis were conducted to assess the relationship between higher and lower levels of GdIgA1, anti-GdIgA1 antibodies, and the proteinuria remission for MCD-IgAN patients. A two-sided *p*-value <0.05 was considered statistically significant. All analyses and graphs were conducted using GraphPad Prism version 8.0 for Windows (GraphPad Software, San Diego, CA, United States) and IBM-SPSS22.0 (IBM-SPSS Inc., Armonk, NY).

## Results

### Baseline Demographic, Clinical, and Pathological Characteristics

A total of 27 MCD-IgAN patients with a mean age of 30.6 ± 12.1 years old were enrolled in this study. The mean age of MCD-IgAN patients was comparable to that in another Chinese study (Li et al., 2016) whereas lower than the median age of 46 in one study from United States (Herlitz et al., 2014). The cohort included 17 men and 10 women. The male proportion of MCD-IgAN patients was 63%, which was comparable to those in previous studies. Twenty-five patients fulfilled criteria for NS, and the remaining two patients had nephrotic range proteinuria without hypoalbuminemia (Alb 32.2 and 31.8 g/L). Compared to IgAN group, patients with MCD-IgAN had significantly higher levels of proteinuria (4.26 ± 1.81 vs. 2.12 ± 1.73 g/24 h, *p* < 0.001) and estimated glomerular filtration rate (eGFR) (120.8 ± 24.3 vs. 81.1 ± 31.5 ml/min, *p* < 0.001) while lower levels of albumin (20.0 ± 6.2 vs. 36.9 ± 6.6 g/L, *p* < 0.001), urine blood cells [10.9 (4.4, 32.4) vs. 64.2 (22.5, 194.1)/µl, *p* < 0.001], SBP (116.7 ± 16.5 vs. 131.2 ± 19.9 mmHg, *p* = 0.001) and lower percentages of C3 deposition (66.7 vs. 94.1%, *p* = 0.001), Oxford M1 (37 vs. 89.7%, *p* < 0.001), E1 (0 vs. 17.6%, *p* = 0.017), S1 (0 vs. 75%, *p* < 0.001), and T1/T2 (0 vs. 39.7%, *p* < 0.001) scores. All MCD-IgAN patients have initially received corticosteroids, of which 12 cases were treated in combination with other immunosuppressants (see Table 1).

**TABLE 1:** The baseline clinical and pathological characteristics of MCD-IgAN and typical IgAN patients.

Characteristics	MCD-IgAN (*n* = 27)	IgAN (*n* = 68)	*p*-value
Age (yr; mean ± SD)	30.6 ± 12.1	35.7 ± 12.1	0.063
Male sex, *n* (%)	17 (63.0)	39 (57.4)	0.651
Baseline SBP (mmHg; mean ± SD)	116.7 ± 16.5	131.2 ± 19.9	0.001
Baseline DBP (mmHg; mean ± SD)	81.4 ± 10.7	88.1 ± 14.5	0.033
Baseline proteinuria (g/day; mean ± SD)	4.26 ± 1.81	2.12 ± 1.73	<0.001
Urine red blood cell counts (/µl)	10.9 (4.4, 32.4)	64.2 (22.5, 194.1)	<0.001
Serum creatinine (μmol/L; mean ± SD, median, IQR)	64.3 ± 19.0	95(68, 128.8)	<0.001
eGFR (ml/min/1.73 m^2^; mean ± SD)	120.8 ± 24.3	81.1 ± 31.5	<0.001
CKD stage 1, *n* (%)	25 (92.6)	31 (45.6)	0.001
CKD stage 2, *n* (%)	2 (7.4)	15 (22.1)	
CKD stage 3, *n* (%)	0 (0)	16 (23.5)	
CKD stage 4, *n* (%)	0 (0)	4 (5.9)	
CKD stage 5, *n* (%)	0 (0)	2 (2.9)	
Serum albumin (g/L; mean ± SD)	20.0 ± 6.2	36.9 ± 6.6	<0.001
Hemoglobin (g/L; mean ± SD)	151.0 ± 18.9	133.7 ± 26.0	0.002
Oxford classification of IgAN, *n* (%)			
M1	10 (37)	61 (89.7)	<0.001
E1	0 (0)	12 (17.6)	0.017
S1	0 (0)	51 (75.0)	<0.001
T1/T2	0 (0)	27 (39.7)	<0.001
C1/2	0 (0)	21 (31.3)	0.004
IgG deposition *n* (%)	1 (3.7)	6 (8.8)	0.389
IgM deposition *n* (%)	17 (63)	29 (42.6)	0.096
C3 deposition *n* (%)	18 (66.7)	64 (94.1)	0.001
Follow-up (mo; mean ± SD)	11.1 ± 8.5	14.3 ± 7.3	0.080
RAS blocker treatment, *n* (%)	8 (29.6)	46 (67.6)	0.001
Combined with corticoids and/or other immunosuppressants, *n* (%)	27 (100)	23 (33.8)	<0.001

### Plasma IgA1, Gd-IgA1, and IgG Antiglycan Autoantibodies

**FIGURE 1:**
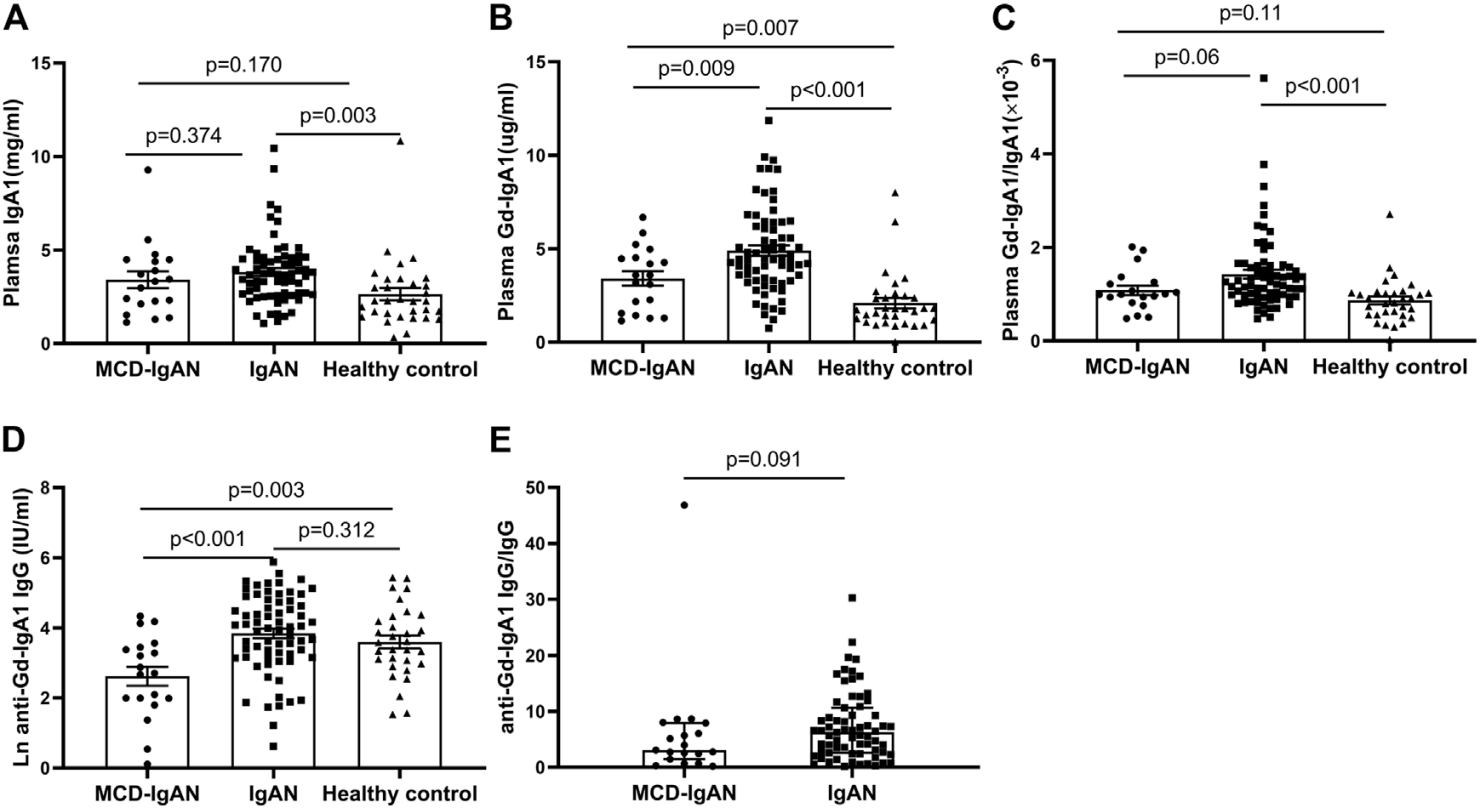
Levels of plasma Gd-IgA1 and its autoantibodies in MCD-IgAN, IgAN, and healthy controls. Patients of MCD-IgAN had comparable levels of IgA1 with those in IgAN patients **(A)**. Compared to IgAN patients, MCD-IgAN patients had both lower levels of plasma GdIgA1 **(B)** and its autoantibodies (anti-GdIgA1 IgG) **(D)**. In addition, significant higher levels of GdIgA1 and its autoantibodies were found in MCD-IgAN patients than healthy controls **(B**,**D)**. After adjusted by the total IgA1 levels, total IgG levels, the same trend of GdIgA1 and its autoantibodies were seen between MCD-IgAN and IgAN groups **(C**,**E)**.

Levels of plasma IgA1, Gd-IgA1, and IgG autoantibodies against GdIgA1 were detected in 19 MCD-IgAN patients and 68 IgAN patients with blood samples available. As shown in Figure 1A, the levels of total IgA1 in IgAN group was significantly higher compared to those in healthy controls (3.82 ± 1.73 vs. 2.65 ± 1.87 mg/ml, *p* = 0.003), but with a comparable level in patients with MCD-IgAN patients (3.82 ± 1.73 vs. 3.41 ± 1.93 mg/ml, *p* = 0.374). However, MCD-IgAN patients had lower levels of GdIgA1 than those in IgAN patients (3.41 ± 1.68 vs. 4.92 ± 2.30 µg/ml, *p* = 0.009) and higher GdIgA1 levels than those in healthy controls (3.41 ± 1.68 vs. 2.10 ± 1.58 µg/ml, *p* = 0.007) (Figure 1B). After adjusting the total IgA1 concentration, there was a similar trend for Gd-IgA1/IgA1 levels with unadjusted GdIgA1 levels among the three groups (Figure 1C).

The plasma levels of IgG antiglycan autoantibodies targeting the GdIgA1 in patients with IgAN were significantly higher than those in patients with MCD-IgAN (76.58 ± 71.22 vs. 23.25 ± 22.59 IU/ml, *p* < 0.001) (Figure 1D). In contrast, no significant difference in IgG autoantibodies levels between IgAN patients and healthy controls were found. After the adjusting serum IgG level, a consistent trend for IgG autoantibodies/IgG between MCD-IgAN and IgAN group was found (Figure 1E).

### Glomerular Deposition of IgA and Gd-IgA1

**FIGURE 2:**
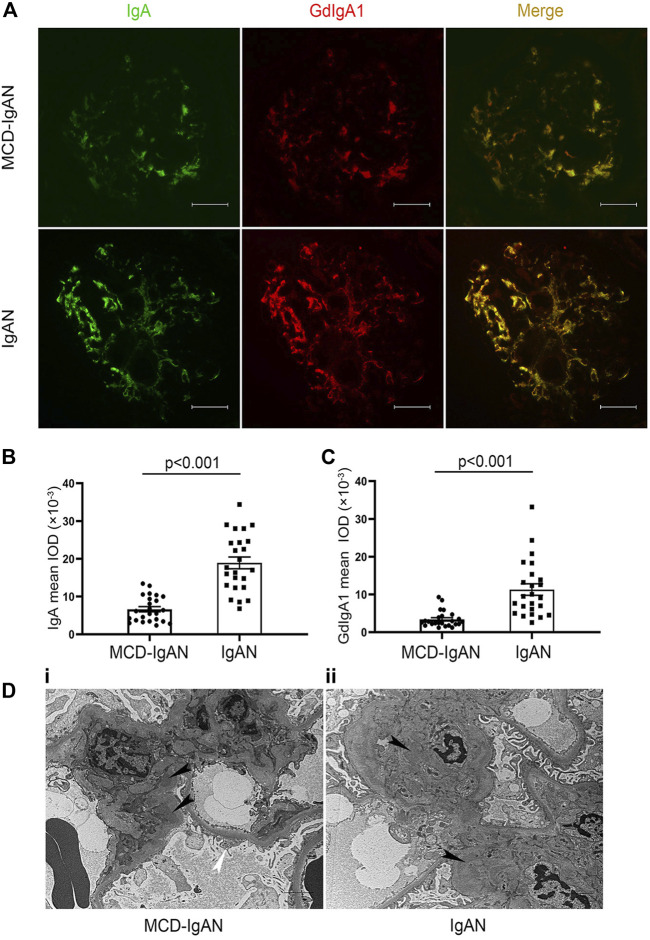
Immunofluorescence (IF) staining of IgA and Gd-IgA1 in the glomerulus of MCD-IgAN and non-MCD-IgAN patients. **(A)** IgA (green, left panel) and GdIgA1 (red, middle panel) deposition were shown in IgAN-MCD patients **(top panel)** and IgAN patients **(bottom panel)**. IF signals of both IgA and GdIgA1were mainly concentrated in the mesangial regions of glomerulus and were co-deposited (yellow, right panel). In contrast to IgAN, weaker signals of both IgA and GdIgA1 were found in glomerulus of patients with MCD-IgAN. Significantly lower mean fluorescent optical density of IgA **(B)** and GdIgA1 **(C)** in the glomerulus were found in MCD-IgAN patients (*n* = 24) compared to IgAN patients (*n* = 24). Scale bar = 50 µm. **(D)** Typical pathological change in glomeruli under electron microscopy observation in MCD-IgAN and non-MCD-IgAN patients were shown. Examples of dense deposits in the mesangial region (black arrow) and diffuse podocyte foot process effacement were observed in the glomeruli of one MCD-IgAN patient **(i)**. Example of dense deposits was found in the mesangial region of one IgAN patient **(ii)**.

Because of the significant difference in plasma GdIgA1 levels between MCD-IgAN and IgAN patients, available paraffin-embedded kidney tissues from 24 MCD-IgAN patients were prepared for double IF staining of IgA and Gd-IgA1. Renal tissues from 24 age- and gender-matched IgAN patients were selected as controls (Supplementary Table S1). Gd-IgA1 was distributed mainly in the mesangial area with co-deposits of IgA in the kidney tissue of MCD-IgAN patients, similar to those in IgAN patients (Figure 2A). Nevertheless, weaker fluorescence intensities of both IgA and GdIgA1 were observed in the kidney of MCD-IgAN patients compared to those in IgAN patients, as shown by the analysis of mean optical density per glomerular compartment in Figures 2B,C (mean optical density of IgA, 18.9 ± 7.6 vs. 6.6 ± 3.4 × 10^−3^, *p* < 0.001, and GdIgA1, 11.5 ± 8.0 vs. 3.3 ± 2.2 × 10^−3^, *p* < 0.001, IgAN and MCD-IgAN, respectively). Under electron microscopy observation, dense deposits in the mesangial region (black arrow) were shown in the kidney tissue of both MCD-IgAN and IgAN patients, while diffuse podocyte foot process effacement (white arrow) was only observed in the glomeruli of MCD-IgAN patients (Figure 2D).

### GdIgA1 and its Autoantibodies on Proteinuria Remission

**FIGURE 3:**
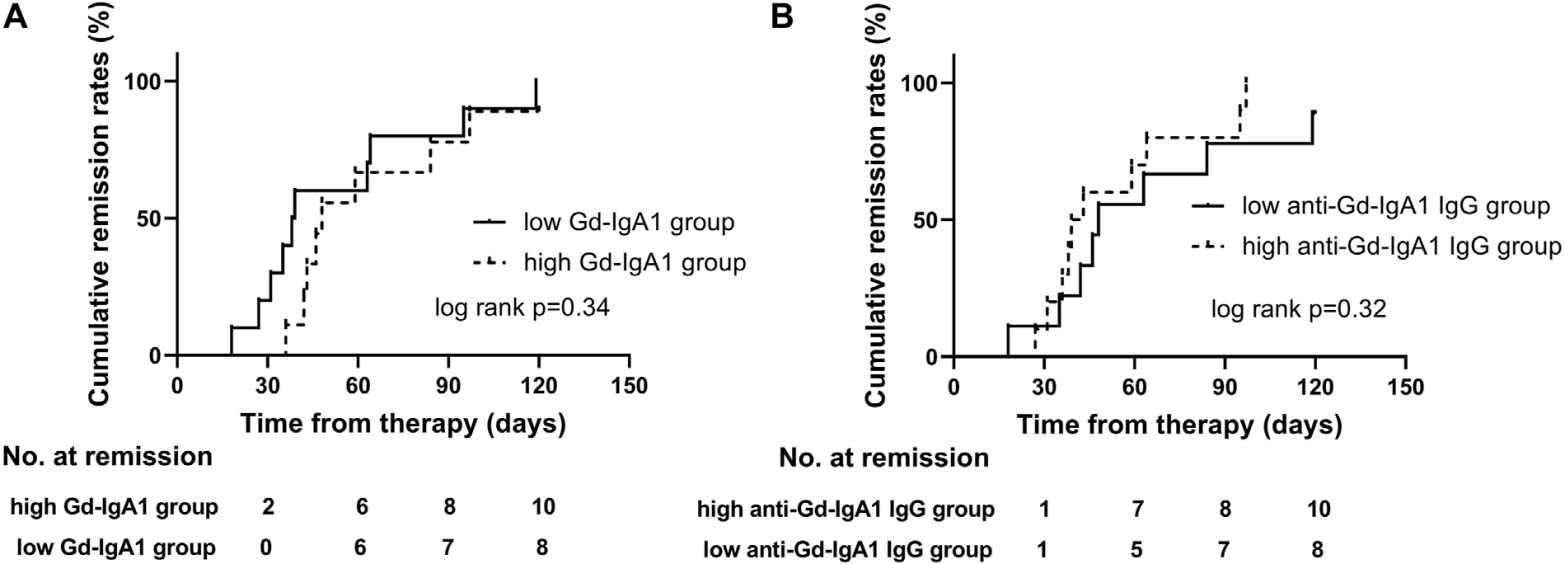
First remission in MCD-IgAN patients with different stratification of plasma galactose-deficient IgA1 (Gd-IgA1) levels **(A)** and IgG antibodies against anti-GdIgA1 **(B)**. There were no significant differences in protein remission in groups of MCD-IgAN patients with higher versus lower GdIgA1 concentrations, and groups of higher versus lower anti-GdIgA1 antibodies.

High risk of progression in IgAN is currently defined as proteinuria >1 g/24 h after therapy in the KDIGO guideline. Furthermore, higher levels of GdIgA1 and its autoantibodies are associated with poor renal outcomes in IgAN patients (Berthoux et al., 2012; Zhao et al., 2012). Here, we tried to analyze the relationship of proteinuria remission with different levels of GdIgA1 or antiglycan antibodies. Among all included patients, sixty-one cases of IgAN patients and twenty-six cases of MCD-IgAN patients were retrospectively followed for 14.3 ± 7.3 and 11.1 ± 8.5 months, respectively. Five patients (5/61, 8.2%) of the IgAN patients reached to end-stage of kidney disease or more than 30% decrease in eGFR, while none of the MCD-IgAN patients reached above kidney endpoint, and all had stable renal function at the last follow-up. Twenty-five MCD-IgAN patients achieved complete remission (CR) of proteinuria during the follow-up periods; among them, four patients experienced relapsing proteinuria. Kaplan–Meier survive analysis was conducted to estimate different levels of GdIgA1 or its autoantibodies on the effect of CR in MCD-IgAN patients. The results showed neither higher levels of GdIgA1 (log rank *p* = 0.34, Figure 3A) nor its antiantibodies (log rank *p* = 0.32, Figure 3B) were associated with the time to first CR within MCD-IgAN patients.

### Poly-IgA1 Complexes Purified From MCD-IgAN Patients Had Lower Inflammatory Effect

**FIGURE 4:**
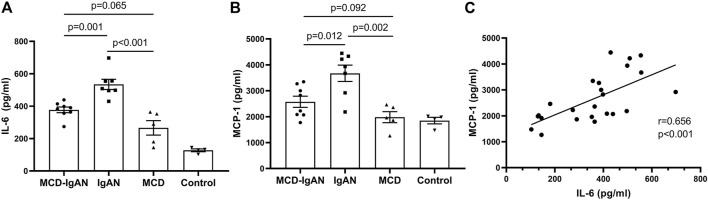
Secreted IL-6 and MCP-1 by cultured human mesangial cells treated with same dosage of ploly-IgA1 from MCD-IgAN, IgAN and MCD patients. **(A)** Higher levels of IL-6 were detected in supernatants of cultured mesangial cells treated with poly-IgA1 from IgAN patients (*n* = 7) than those from MCD-IgAN (*n* = 8) and MCD patients (*n* = 5), while non-significant difference in IL-6 levels in supernatants of cultured mesangial cells treated with poly-IgA1 from MCD-IgAN or MCD patients were found. **(B)** Consistent results of MCP-1 levels among three groups were shown. **(C)** Positive relationships of secreted MCP-1 and IL-6 levels in mesangial supernatants with stimulation of poly-IgA1 were observed.

Plasma poly-IgA1 from eight MCD-IgAN patients, seven non-MCD-IgAN patients, and five MCD patients were purified to stimulate cultured human mesangial cells. Clinical characteristics of these patients were described in Supplementary Table S2. Supernatants of treated mesangial cells were collected for IL-6 and MCP-1 detection. We found that poly-IgA1 complex derived from MCD-IgAN patients, IgAN patients, and MCD patients upregulated the excretion of the mesangial cell inflammatory cytokines MCP-1 and IL-6 compared to PBS control. Poly-IgA1 complexes from MCD-IgAN patients induced lower expression of mesangial MCP-1 and IL-6 compared to those from IgAN (MCP-1: 2,574.4 ± 607.5 vs. 3,671.8 ± 835.5 pg/ml, *p* = 0.012; IL-6: 377.3 ± 49.5 vs. 534.3 ± 83.6 pg/ml, *p* = 0.001, Figures 4A,B) and higher levels of MCP-1 and IL-6 than those in MCD group (MCP-1: 2,574.4 ± 607.5 vs. 1,981.8 ± 474.1 pg/ml, *p* = 0.092; IL-6: 377.3 ± 49.5 vs. 266.3 ± 99.5 pg/ml, *p* = 0.065; Figures 4A,B). Moreover, levels of MCP-1 in supernatants were positively correlated with IL-6 (correlation coefficient = 0.656, *p* < 0.001; Figure 4C). The results indicated that poly-IgA1 from MCD-IgAN patients had weaker inflammatory stimulation to mesangial cells than those from IgAN patients, which was consistent with lower urinary MCP-1/creatinine levels in MCD-IgAN than IgAN patients (Supplementary Figure S2).

## Discussion

Whether MCD-IgAN is a variant form of IgAN or simply MCD superimposed on mild underlying IgAN or even with quiescent IgA deposition coincidence of MCD has not been established yet. Although several published studies have described the clinical and pathological features of MCD-IgAN and suggested it as a dual glomerulopathy (Qin et al., 2013; Wang et al., 2013; Woo et al., 2013; Herlitz et al., 2014; Li et al., 2016; Cho et al., 2020), advanced studies on the pathogenesis of MCD-IgAN are rare. In the present study, we explored the comprehensive immune characteristics of MCD-IgAN patients. Compared to IgAN patients, MCD-IgAN patients had lower levels of plasma GdIgA1, IgG antiglycan autoantibodies, and weaker fluorescence intensities of GdIgA1 in the glomerulus than those in IgAN patients. *In vitro*, the inflammatory response of the mesangial cells (IL-6 and MCP-1 production) to poly-IgA1 complex derived from MCD-IgAN patients was significantly lower than that from IgAN participants. Our findings support MCD-IgAN as a dual glomerulopathy, namely, mild IgAN with superimposed MCD from the pathogenesis point of view.

Current studies suggested the development of IgAN as four-hit processes with aberrant glycosylation of IgA1, production of antibodies directed against galactose-deficient IgA1, formation of immune complexes, and accumulation of these complexes in the glomerular mesangium to initiate renal injury (Suzuki et al., 2011). However, with respect to MCD-IgAN, whether it shares the same immunopathogenesis of the four-hit processes as IgAN still needs to be investigated. Until now, only one published study from Cho et al. confirmed positive Gd-IgA1 deposition in the glomeruli of IgAN-MCD patients and inferred that Gd-IgA1 played a role in the pathogenesis of MCD-IgAN and IgAN (Cho et al., 2020). However, this information should be interpreted carefully. First, the frequency of mesangial Gd-IgA1 deposition in healthy donors was as high as 13%–26% (Suzuki et al., 2003; Nakazawa et al., 2019), suggesting quiescent IgA1 deposition was not uncommon (Suzuki et al., 2003; Nakazawa et al., 2019). Secondly and importantly, the glycosylation aberrancy of IgA1 alone is not sufficient to induce renal injury, the autoantibodies levels and biological effects of poly-IgA1 from MCD-IgAN are unknown in Cho’s study. Therefore, in this study, we conducted the first comprehensive study and explored the multiple proposed factors that might explain the pathogenesis of MCD-IgAN, including levels of Gd-IgA1, antiglycan antibodies, deposition of Gd-IgA1, and corresponding proinflammatory effects of the poly-IgA1complexes. We found that MCD-IgAN patients had lower circulating Gd-IgA1 and IgG glycan-specific antibodies, weaker GdIgA1 deposits than age- and gender-matched IgAN patients but higher plasma GdIgA1 than healthy controls. Moreover, purified poly-IgA1 complexes from MCD-IgAN induced weaker effects on mesangial inflammatory cytokines production than those from IgAN while higher cytokine levels than those from MCD. These findings may be related to the relatively indolent nature of IgA deposits, not as pathophysiological as IgAN patients, and mild histological lesions with lower Oxford scores in MCD-IgAN, supporting the lack of typical manifestations of IgAN. Taken together, this study provides immunological evidence supporting that MCD-IgAN represents a dual glomerulopathy, namely, mild IgAN with superimposed MCD, and should be treated in accordance with the guidelines for MCD.

In this study, we found higher Gd-IgA1 but lower anti-GdIgA1 IgG levels in plasma of MCD-IgAN patients compared to that in healthy controls (Figures 1B,D). These were because of the higher sensitivity and specificity of GdIgA1 than anti-GdIgA1 antibodies in IgAN. Previous studies indicated that higher levels of IgG autoantibodies were only found in IgAN patients with the highest risk for dialysis or death (Tomana et al., 1999; Moldoveanu et al., 2007; Berthoux et al., 2012), and the MCD-IgAN patients we included were at lower risk for dialysis. Furthermore, in MCD-IgAN patients, due to the loss of IgG from kidney filtration, serum total IgG (5.6 ± 3.5 g/L) was lower than that in healthy controls (7–16 g/L), as well as the IgG autoantibody levels.

Previous studies had demonstrated that the deposited IgA in the glomerulus of IgAN was galactose deficient (Allen et al., 2001; Berthelot et al., 2015; Yasutake et al., 2015), and GdIgA1 but not normal glycosylated IgA1 had high propensity to form complexes with antiglycan IgG antibodies (Suzuki et al., 2009). The contents of serum GdIgA1 play an important role in renal deposition other than total IgA. In our study, we found higher GdIgA1 levels in IgAN patients than MCD-IgAN, which was prone to deposit in kidney. Furthermore, the antibody (antihuman Ig alpha heavy chain) we used to detect IgA deposition in the kidney can also recognize GdIgA1, so our results showed an increase in IgA deposition in IgAN patients than that in MCD-IgAN patients (Figure 1B) despite no difference in plasma IgA1 levels between these groups.

Although in this study we investigated the pathogenesis of MCD-IgAN, there were still several limitations. First, due to the low incidence of MCD-IgAN, this was a small single-center study with limited sample size. Second, because of the lack of appropriate animal models of IgAN and MCD-IgAN, merely *in vitro* experiments were conducted to evaluate the proinflammatory effects of IgA1 complexes from MCD-IgAN.

In conclusion, this is the first study to present a comprehensive and precise profile of immune characteristics in MCD-IgAN participants. The results strongly supported that MCD-IgAN cases represent a dual glomerulopathy, namely, mild IgAN with superimposed MCD, and provided robust evidence for the corticosteroids therapy in MCD-IgAN patients as the guidelines recommended.

## Data Availability

The raw data supporting the conclusion of this article will be made available by the authors, without undue reservation.
