# Supplementary material for: Immune Characteristics of IgA Nephropathy With Minimal Change Disease
Source: Front Pharmacol. 2021 Dec 16;12:793511. doi: 10.3389/fphar.2021.793511 (PMC8716750; doi:10.3389/fphar.2021.793511)
Supplement: Supplementary file 1 [file DataSheet1.docx]

**Supplementary Information:**

**Immune characteristics of IgA nephropathy with minimal change disease**

By Huixian Li, Wanhong Lu, Haiyun Li, Xiaoling Liu, Xue Zhang, Liyi Xie, Ping Lan, Xiaoyang Yu, Yinjuan Dai, Xinfang Xie, Jicheng Lv

**Methods and materials**

**Plasma GdIgA1 detection**

Plasma Gd-IgA1 levels were quantified by using the Gd-IgA1-specific monoclonal antibody KM55 ELISA Kit (IBL, Naka, Japan) according to the suggested procedure. In detail, fifty microliters of diluted plasma samples (1:150) and standard were added into appropriate wells of a plate immobilized with KM55 at 7.5 ug/ml. After incubation for 1h at room temperature and four times washes, dilution with an HRP-conjugated mouse anti-human IgA1 α1 chain-specific monoclonal antibody was added to every well for 30 min at 37℃. Then the plate was washed five times and colored by TMB solution, and the reaction was stopped by 1 mol/L sulfuric acid (Wako, Osaka, Japan) Serum Gd-IgA1 levels were extrapolated by referring to a standard curve (4-parameter logistic curve fitting) of optical density (OD) (450 nm) and expressed as ug/ml.

**Plasma IgG autoantibodies detection**

F(ab)_2_-HR fragment containing the hinge region (HR) of Gd-IgA1 was used as the autoantigen to detect the levels of plasma IgG autoantibodies specific for Gd- IgA1 by enzyme-linked immunosorbent assay (ELISA). Briefly, plasma monomeric IgA1 from an IgAN patient was purified by Jacalin (Thermo Scientific, USA) affinity chromatography and an S300 gel filtration molecular sieve (General Electric, USA) by the KTA protein purification system (General Electric Company, USA). The IgG and IgM contained were removed by Protein G and Protein M affinity chromatography columns (General Electric, USA). IgA-specific protease HF48 obtained from Neisseria meningitides (ATCC, US) was used to digest the hinge region of IgA1 between amino acids 238 and 239 for leaving most of the hinge region in the Fab-HR-IgA1 molecule. Fab-HR-IgA1 (autoantigen) was finally purified by a Protein L affinity chromatography column (General Electric, USA). High-binding MaxiSorp 96-well plates (Thermo scientific) were coated with 5ug/mL Fab-HR-IgA1 autoantigen in sodium carbonate buffer (pH=9.6) at 4℃ overnight. After washing three times with PBST and blocking with PBST containing 1% bovine serum albumin (BSA) (Sigma Chemical Company, St Louis, MO) for 60 min at 37℃, diluted plasma samples (1:100 in blocking buffer) and standard dilutions ranging from 100 units/ml to 1.5625 units/ml in blocking buffer were added. Then the plates were incubated for 60 min at 37℃. For the measurement of IgG autoantibody, the standards were obtained from the plasma exchange fluid of an IgAN patient, which contained high levels of IgG autoantibodies tested with the autoantigen. The dilution (1:50) of plasma exchange fluid in the first well was defined as 100 units/ml. After four times washes, an alkaline phosphatase-conjugated goat anti-human IgG monoclonal antibody (Sigma, US) was added to each well for 1 h at 37℃. The plates were then developed with an alkaline phosphatase (AP) liquid substrate system. The results were recorded as the net optical absorbance at 405 nm for the IgG autoantibody in an ELISA reader (Bio-Rad 550, Japan). The results for autoantibody levels are expressed as units/ml.

**Supplemental Table1. The baseline clinical and pathological characteristics of IgA nephropathy patients with and without MCD included.**

| Characteristics |  | MCD-IgAN  (n=24) | IgAN  (n=24) |  | P value |
| --- | --- | --- | --- | --- | --- |
| Age (yr; mean ± SD) |  | 30.6±13.9 | 33.9±7.0 |  | 0.313 |
| Male sex, n (%) |  | 16(66.7) | 14(58.3) |  | 0.766 |
| Baseline SBP (mmHg; mean ± SD) |  | 116.3±14.2 | 132.7±22.7 |  | 0.004 |
| Baseline DBP (mmHg; mean ± SD) |  | 80.7±9.6 | 89.0±17.2 |  | 0.046 |
| Baseline proteinuria (g/day; mean ± SD) |  | 4.10±1.67 | 1.85±1.56 |  | <0.001 |
| Urine red blood cell counts (10^6^/ul) |  | 10.9(4.4,32.4) | 64.2(22.5,194.1) |  | <0.001 |
| Serum creatinine (μmol/L; mean ± SD, median, IQR) |  | 62.7±18.1 | 90.0±50.9 |  | 0.019 |
| eGFR (ml/min /1.73m^2^; mean ± SD) |  | 124.9±25.8 | 97.3±31.8 |  | 0.002 |
| CKD stage 1, n (%) |  | 23(95.8) | 16(66.7) |  | 0.07 |
| CKD stage 2, n (%) |  | 1(4.2) | 4(16.7) |  |  |
| CKD stage 3, n (%) |  | 0(0) | 3(12.5) |  |  |
| CKD stage 4, n (%) |  | 0(0) | 1(4.1) |  |  |
| CKD stage 5, n (%) |  | 0(0) | 0(0) |  |  |
| Serum albumin (g/L; mean ± SD) |  | 20.0±6.8 | 38.6±7.0 |  | <0.001 |
| Hemoglobin (g/L; mean ± SD) |  | 151.0±17.8 | 142.2±22.1 |  | 0.134 |
| Oxford classification of IgAN, n (%) |  |  |  |  |  |
| M1 |  | 9(37.5) | 23(98.1) |  | <0.001 |
| E1 |  | 0(0) | 5(20.8) |  | 0.05 |
| S1 |  | 0(0) | 20(83.3) |  | <0.001 |
| T1/T2 |  | 0(0) | 7(29.2) |  | 0.009 |
| C1/2 |  | 0(0) | 6(25) |  | 0.009 |

**Supplemental Table S2. The baseline clinical and pathological characteristics of MCD-IgAN and typical IgAN patients**

| Characteristics | MCD-IgAN  (n=8) | IgAN  (n=7) | MCD  (n=5) | P* |
| --- | --- | --- | --- | --- |
| Age (yr; mean ± SD) | 31.5±12.5 | 41.3±12.3 | 36.2±20.1 | 0.497 |
| Male sex, n (%) | 5(62.5) | 5(71.4) | 3(60) | 0.928 |
| Baseline proteinuria (g/day; mean ± SD) | 4.64±1.65 | 1.22±0.65 | 4.61±1.37 | 0.969 |
| Serum creatinine (μmol/L; mean ± SD, median, IQR) | 65.9±17.1 | 84.8±28.8 | 64.6±26.3 | 0.887 |
| eGFR (ml/min /1.73m2; mean ± SD) | 128.7±24.5 | 89.8±25.8 | 137.2±29.9 | 0.451 |
| Serum albumin (g/L; mean ± SD) | 18.7±6.2 | 37.3±3.9 | 17.0±3.5 | 0.423 |
| Hemoglobin (g/L; mean ± SD) | 159.6±15.5 | 136.3±19.8 | 142.1±28.8 | 0.09 |

***p value between MCD-IgAN and MCD group.**

**
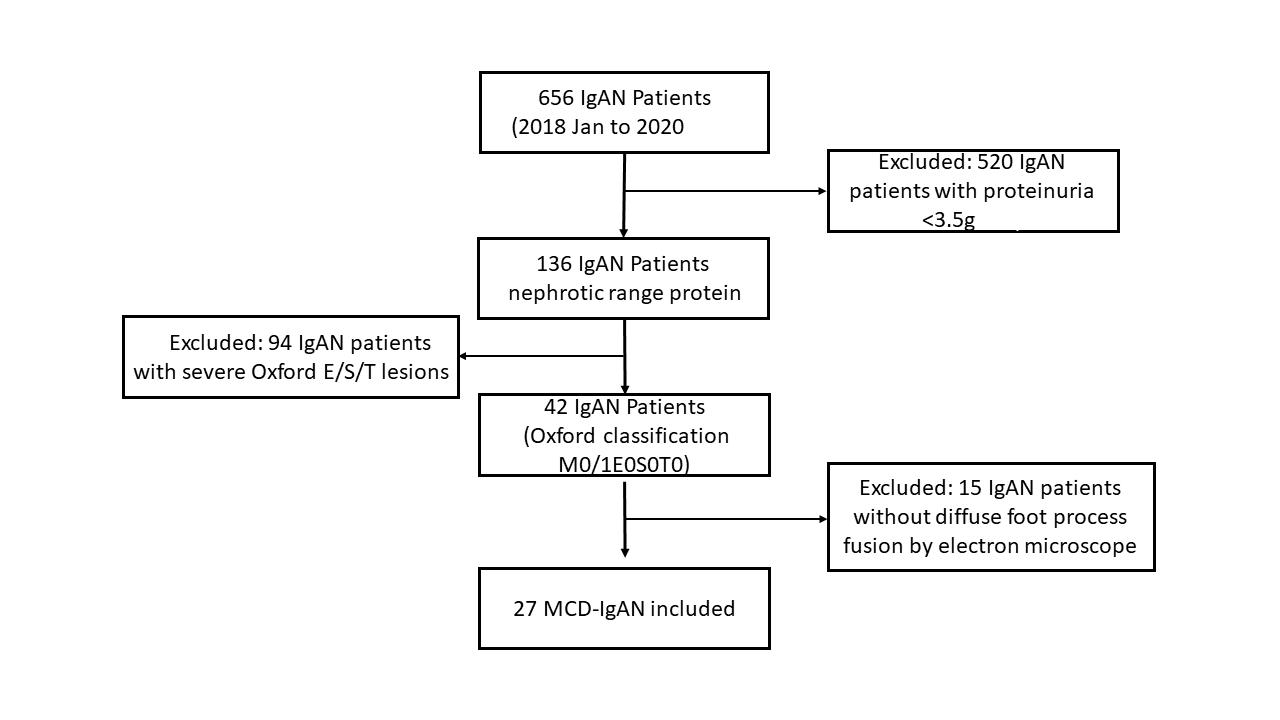
Figure S1. The flowchart of 27 MCD-IgAN patients enrolled in the study.**


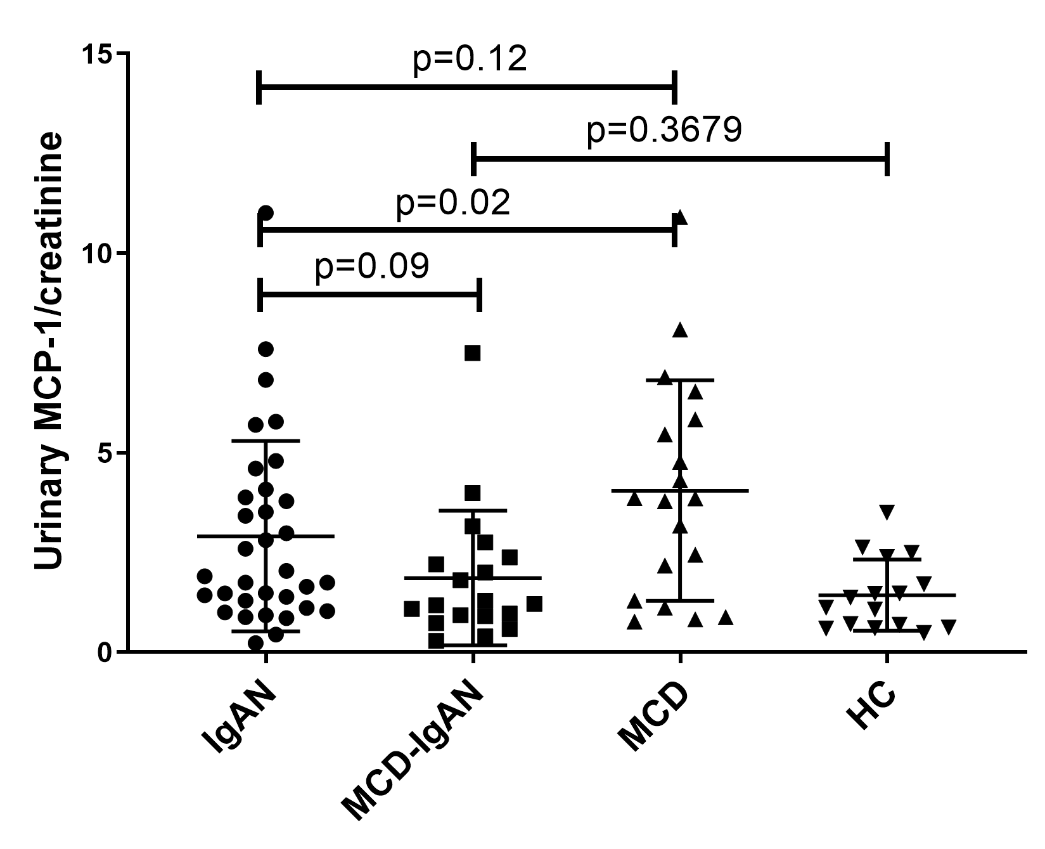


**Figure S2.** Urinary MCP-1/creatinine levels in MCD-IgAN patients, IgAN patients, MCD patients and healthy controls.
